# Complete genome sequences of eight potential biocontrol microorganisms isolated from Washington apple orchards

**DOI:** 10.1128/mra.00779-26

**Published:** 2026-08-13

**Authors:** Tanmoy Mukherjee, Ricardo Delgado Santander, Youfu Zhao

**Affiliations:** 1 Department of Plant Pathology—IAREC, College of Agricultural, Human and Natural Resource Sciences (CAHNRS), Washington State University 6760, Prosser, Washington, USA; University of Strathclyde, Glasgow, United Kingdom

**Keywords:** *Pseudomonas*, *Erwinia*, biocontrol agents, fire blight

## Abstract

Eight potential biocontrol bacteria were isolated from diseased apple (*Malus domestica*) tissues. Here, we report high-quality complete genome sequences for the eight isolates. These data might help in understanding the genetic basis of biocontrol activity, strain compatibility, and ecological adaptation of biocontrol agents for fire blight disease management.

## ANNOUNCEMENT

Fire blight disease, caused by *Erwinia amylovora*, is a serious disease threatening apple and pear production worldwide (1). In 2022, eight strains across seven genera of both gram-positive and gram-negative bacteria were co-isolated with *E. amylovora* from diseased apple (*Malus domestica*) tissues from eastern Washington orchards, as previously described (2). These isolates exhibited strong antagonistic activities against *E. amylovora* (2). Here, we report high-quality complete genomes of the eight strains, including the complete genome of *Pseudomonas parakoreensis* strain 2180, which is closely related to a new species *P. parakoreensis* sp. nov. (BML-PP030^T^), sharing 99.78% average nucleotide identity (ANI) (3).

Pure cultures of isolates stored locally at −80°C in 20% glycerol were streaked on LB agar and incubated at 28°C for up to 72 h to obtain single colonies. Overnight cultures of each strain grown in 10 mL LB broth at 30°C with shaking at 200 rpm were pelleted. High molecular weight genomic DNA (HMW gDNA) was isolated using Qiagen Genomic-tip 20/G. All library preparations and sequencing were performed at SeqCenter (Pittsburgh, PA, USA). For Illumina sequencing, libraries were prepared using the tagmentation and PCR-based Illumina DNA prep kit and sequenced on a NovaSeqX instrument (150 bp paired-end reads). For PacBio sequencing, gDNAs were sheared with a Megaruptor 3 (Diagenode Inc.) (6–10 kb), and library preparation was performed with and without final size selection using the PacBio SMRTbell prep kit 3.0 followed by sequencing on a Revio 25M Flowcell using the Revio Polymerase kit following the manufacturer’s instructions. Default parameters were used for all software unless otherwise specified. Illumina reads were filtered using fastp v0.23.4 (4) (Q25 ≥ 45 bp) after adapter and phiX contamination were removed using bbduk v39.29 (5). PacBio reads were filtered with Chopper v0.9.0 (6) (Q25 ≥ 5,000 bp) and any potential adapter contaminations. Human DNA contamination was removed by aligning reads against the human reference genome GCA_000001405.28_GRCh38.p13, using bowtie2 v2.5.4 (7) and minimap2 v2.28 (8).

Post-cleaning PacBio reads from runs with and without final size selection were then merged and assembled using Autocycler v0.5, which also included plassembler v1.8.0 for recovering plasmid (9, 10). The autocycler consensus assembly was polished using Illumina reads with NextPolish2 v0.2.1 (11), circularized with dnaapler v1.2.0 (12), and repolished. Assembly quality control was evaluated using CheckM2 v1.0.2 (13) and GCI v1.0 (14), while circularity was confirmed by Bandage v0.9.0 (15). The final assembly was annotated using the Prokaryotic Genome Annotation Pipeline (v6.10) (16). The genomes were classified using GTDB-Tk v2.6.0 (17) and the Genome Taxonomy Database (GTDB) (18) full database v226 with default settings. Assembly metrics are summarized in Table 1. These eight genomes provide valuable resources for studying the genetic basis of *E. amylovora* biocontrol and designing effective microbial consortia for sustainable fire blight management.

**TABLE 1:** General information about the bacterial strains and their genome assembly statistics

Identity based on GTDB^*b*^	*Erwinia billingiae*		*Pantoea agglomerans*	*Pseudomonas parakoreensis*	*Pseudomonas syringae*	*Microbacterium* sp.	*Bacillus cereus*	*Curtobacterium* sp.	*Rahnella inusitata*
Strains	2186		ABR1s	2180	ABR5	ABR10	2175	ABR8	EL51
GenBank/genome accession no.	JBSZSZ000000000/GCA_057812255.1		JBSZSY000000000/GCA_057812235.1	JBSZSX000000000/GCA_057812215.1	JBSZSW000000000/GCA_057812195.1	JBSZSV000000000/GCA_0578121 75.1	JBSZSU000000000/GCA_057812155.1	JBSZST000000000/GCA_0578121 25.1	JBSZSS000000000/GCA_057812115.1
BioSample	SAMN50736746		SAMN50736747	SAMN50736748	SAMN50736749	SAMN50736750	SAMN50736751	SAMN50736752	SAMN50736753
SRA^*a*^	SRS27525408		SRS27525409	SRS27525410	SRS27525411	SRS27525412	SRS27525413	SRS27525414	SRS27525415
Closest genome^*c*^	GCF_000196615.1		GCF_001598475.1	GCF_021602155.1	GCF_000507185.2	GCF_001424225.1	GCF_000007825.1	GCF_019844075.1	GCF_003263515.1
ANI^*d*^	98.7		98.5	99.8	97.9	98.2	97.3	97.7	99
GC%	55.1		55	60.1	59.1	69.8	34.9	70.8	53.1
PacBio run 1 (read, N50)^*e*^	200,665, 13,704		222,200, 12,541	198,366, 15,122	89,498, 14,092	91,045, 13,778	175,069, 11,875	67,321, 7,980	145,462, 12,878
PacBio run 2 (read, N50)^*f*^	173,938, 13,716		448,283, 13,050	191,692, 15,237	89,952, 14,181	90,834, 14,258	301,924, 12,307	48,778, 9,757	106,340, 13,055
Merged PacBio reads post-filtering (read, N50)^*g*^	257,940, 13,632		381,377, 13,006	248,806, 15,005	126,613, 14,004	88,320, 13,899	284,834, 12,515	32,890, 10,572	153,871, 13,042
Illumina read pairs raw (post-filtering)^*h*^	6,249,360 (5,664,921)		6,061,394 (5,507,055)	7,544,154 (6,723,378)	6,247,478 (5,557,004)	5,402,601 (4,457,652)	4,796,072 (4,206,554)	6,876,254 (5,706,097)	5,974,143 (5,330,114)
PacBio coverage^*i*^	662		896	564	289	313	676	84	387
Total no. of contigs	3		5	2	2	2	12	1	2
Total genome size	5,109,160		5,049,100	6,553,766	6,008,749	3,658,953	6,357,730	3,951,737	4,924,327
Chromosome size	4,915,693		4,059,045	6,495,978	5,935,665	3,606,731	5,266,614	3,951,737	4,409,376
Plasmid sizes	97,945, 95,522		192,849, 111,294, 142,146, 543,766	57,788	73,084	52,222	487,450, 300,458, 91,406, 75,441, 69,062, 17,064, 12,149, 8,513, 7,921, 7,635, 14,017	NA^*l*^	514,951
Assembly N50	4,915,693		4,059,045	6,495,978	5,935,665	3,606,731	5,266,614	3,951,737	4,409,376
GCI (genome continuity inspector)^*j*^	99.9996		99.9995	99.9997	99.9996	99.9994	99.9994	99.9995	99.9995
CheckM2^*k*^									
Completeness model	General	99.5	100	99.44	99.08	99.38	100	99.23	100
	Specific	100	100	100	100	99.98	100	99.99	100
Contamination level	0.28		0.01	0.26	0.19	0.05	0.02	0.17	0.02

^*a*^ The sum of PacBio runs 1 and 2 (total spots).

^*b*^ Identification of the isolates and their taxonomic assignment was carried out using the GTDB (15, 16).

^*c*^ The reference genome identified by GTDB as most similar to the query genome.

^*d*^ Average nucleotide identity between the query genome and the corresponding reference genome.

^*e*^ The total raw reads, N50 from the first PacBio run, without size selection.

^*f*^ The total raw reads, N50 from the second PacBio run, with size selection.

^*g*^ The combined PacBio reads from both runs after quality and length filtering with the read N50 shown in parentheses.

^*h*^ The total raw Illumina reads, with the number remaining after quality and length filtering shown in parentheses.

^*i*^ The genome coverage calculated using the merged, post-filtering PacBio reads.

^*j*^ The genome completeness score reported by GCI.

^*k*^ Genome completeness using two models, general and species-specific model, along with the contamination level.

^*l*^ NA, not applicable.

## Data Availability

This Whole Genome Shotgun (WGS) project under BioProject number PRJNA1309151 and SRA number SRP656842 has been deposited at GenBank under the accessions JBSZSS000000000 to JBSZSZ000000000. The version described here is the first version. The links for all WGS and genome assembly data can be found in Table 1.
